# Short-term monocular occlusion produces changes in ocular dominance by a reciprocal modulation of interocular inhibition

**DOI:** 10.1038/srep41747

**Published:** 2017-02-02

**Authors:** Eva Chadnova, Alexandre Reynaud, Simon Clavagnier, Robert F. Hess

**Affiliations:** 1 McGill Vision Research, Dept. Ophthalmology, McGill University, Montreal, Quebec, Canada

## Abstract

Ocular dominance can be modulated by short-term monocular deprivation. This changes the contribution that each eye makes to binocular vision, an example of adult cortical neuroplasticity. Optical imaging in primates and psychophysics in humans suggest these neuroplastic changes occur in V1. Here we use brain imaging (MEG) in normal adults to better understand the nature of these neuroplastic changes. The results suggest that short-term monocular deprivation, whether it be by an opaque or translucent patch, modulates dichoptic inhibitory interactions in a reciprocal fashion; the unpatched eye is inhibited, the patched eye is released from inhibition. These observations locate the neuroplastic changes to a level of visual processing where there are interocular inhibitory interactions prior to binocular combination and help to explain why both binocular rivalry and fusional tasks reveal them.

It is well known that adults can exhibit a degree of ocular dominance plasticity that is reflected in the strengthening of the undeprived eye relative to that of the deprived eye following prolonged periods (in the order of days) of monocular deprivation (for review, see ref. 1). More recently another form of ocular dominance plasticity has been described in adults following very short periods of monocular deprivation in which the opposite effects occur, namely the deprivation results in the patched eye becoming stronger and the unpatched eye weaker. This short-term monocular deprivation (2–3 hrs) in normal adults leads to changes in ocular dominance that lasts for up to 30 minutes. This was first shown using a binocular rivalry paradigm, with the previously patched eye dominating in the perceptual alternations after the patch is removed^2^. Zhou *et al*.^3^, using a task that involved assessing the relative contributions of each eye to the binocularly fused percept, demonstrated that the ocular dominance is shifted in favour of the previously patched eye and that this is reciprocal in nature; the unpatched eye’s contribution declines while the patched eye’s contribution increases once the patch is removed. A similar reciprocal influence has been observed in the primate using instrinsic optical images of ocular dominance columns in the visual cortex^4^. These data from optical imaging together with data from human psychophysics using a more controlled monocular deprivation involving dichoptic movies^5^ suggests that these neuroplastic changes are binocular in nature and involve an early stage of processing in V1.

Models of binocular vision incorporate separate inhibitory and excitatory pathways^6^,^7^. In both the Ding and Sperling^6^ and Meese *et al*.^7^ models there is a stage of reciprocal inhibition prior to excitatory combination of signals. A plausible explanation for why these neuroplastic changes in ocular dominance can be revealed using binocular rivalry of non-fusible stimuli as well as more typical combination tasks involving fusible stimuli might be because they occur at the level of these dichoptic inhibitory interactions. This follows from the close association between binocular rivalry and dichoptic masking^8^,^9^.

In a recent psychophysical study using the novel method developed by Georgeson and Wallis^10^ of how binocular interactions are affected by short term deprivation, it was found that the main effect of the deprivation was to alter the suppressive balance between the eyes^11^, adding further support to the hypothesis that short term deprivation specifically alters ocular dominance by modulating the contralateral inhibitory balance between the eyes prior to binocular combination.

Here we employ a more direct approach using MEG to investigate the nature of the neuroplastic changes underlying ocular dominance plasticity. We use a frequency-tagging approach to identify the signals from each eye combined with a monocular and dichoptic stimulation to reveal how the modification of information in the primary visual cortex results in short term changes in ocular dominance. The results suggest these neuroplastic changes occur at a dichoptic site and involve interocular reciprocal changes in inhibition.

## Methods

### Participants

All subjects underwent a standard optometric examination and all had normal acuity and normal binocular vision. All subjects were right handed. We collected the data from eight subjects (1 female, 7 males, age: 29.4 + /− 4.6, using the opaque patch protocol, and four male subjects who were also part of the opaque protocol group (age: 32.5 + /− 4.8) using the translucent patch protocol. Two sessions of translucent patch data were collected and pooled.

This research has been approved by the Research Ethics Board of the Montreal Neurological Institute, consistent with the tenets of the Declaration of Helsinki. Informed consent was obtained from the subjects after explanation of the nature and possible consequences of the study.

### Stimuli

**Figure 1:**
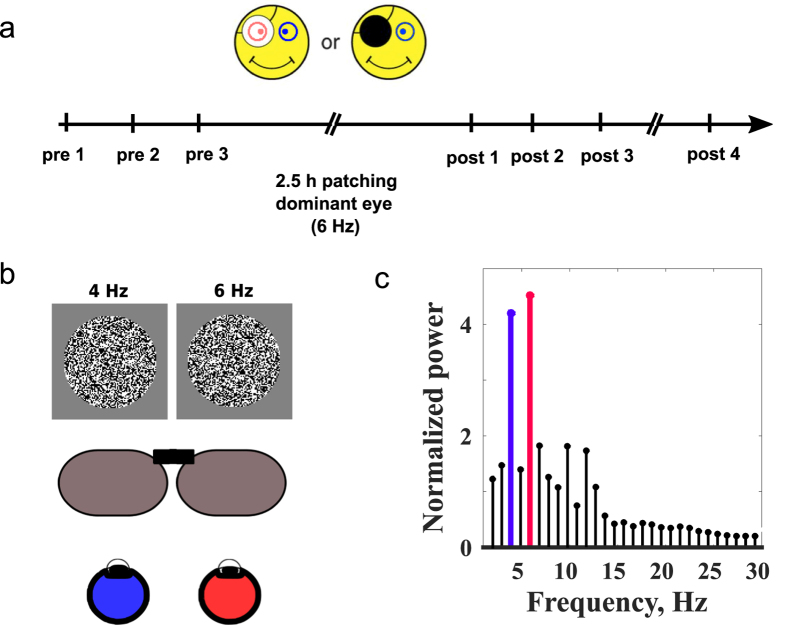
Procedures. (**a**) The time line showing three baseline measurements, a 2.5 hr period of monocular deprivation using either a black patch or a translucent patch followed by 4 measurements are various times after removal of the patch. (**b**) The stimulus and its dichoptic presentation using polarized glasses and its temporal frequency tagging. (**c**) The response frequency spectrum and the temporal frequency tagged components representing the responses of each eye.

The experimental presentation was coded using the Psychophysics toolbox^12^,^13^ in Matlab. A visual stimulus consisted of binary noise pattern with a soft circular window presented dichoptically (Fig. 1). We used a steady state visually evoked responses (SSVER) paradigm^14^ with the stimuli projected to each eye at stimulus onset/offset temporally sinusoidaly modulated from noise pattern to mean luminance at frequencies of 4 Hz and 6 Hz, respectively (Fig. 1c).

The trial duration was 4 seconds, with a 1.5-second inter-trial interval. The stimulus sustained 8 degrees of visual field. The contrast of the stimuli was set to 32% or 0% depending on the condition tested (monocular dominant eye, monocular non-dominant eye, dichoptic or mean luminance). A static black fixation cross was presented at the center of the visual field and was present at all times. Eye dominance was assessed using the porta test^15^. We used a 60 Hz refresh rate gamma-corrected 3D LG monitor (23″, 1920 × 1080, active area 509 × 290 mm) with a set of polarizers for dichoptic stimulation (Fig. 1b). The monitor was placed 170 cm away from the observer in a dark magnetically shielded MEG room.

### Procedure

Figure 1A shows the timeline of the patching protocol. Three recording blocks were performed before the patch was applied. A recording block consisted of 20 repetitions of the 4 possible contrast arrangements in the two eyes for a duration of approximately 8 minutes. The four conditions included*: monocular stimulation of 4* *Hz tagged eye* (non-dominant) with 32% contrast with the contrast in the other eye set at 0%, *monocular stimulation of the 6* *Hz tagged eye* (dominant) eye with 32% contrast and contrast in the other eye set to 0%, *dichoptic stimulation* with contrast to 4 and 6 Hz tagged eyes set to 32% and finally the *mean luminance condition* with the contrast set to 0% to both 4 and 6 Hz tagged eyes (mean luminance). The patch was then applied over the dominant eye (for standardization), and participants were allowed to leave the recording booth for 2.5 hours and were instructed to stay awake, not engage in visually demanding tasks and keep their two eyes open. Upon return, we completed three recording runs following the same steps as in the pre-patch. The first post-patching block (P1) was recorded immediately upon patch removal, this was followed by blocks P2 and P3, with each block lasting approximately 8 minutes. The final P4 segment was recorded 45 minutes after the removal of the patch.

### MEG data acquisition

The recordings began with a 2-minute empty-room MEG recording, to capture daily environmental noise statistics (sample data covariance across MEG channels) that were later used for MEG source modeling.

MEG data were collected using a CTF OMEGA System with 275 axial gradiometers, inside a 3-layer magnetically shielded room. A Polhemus Isotrak system was used to digitize the participants’ fiducial landmarks (nasion and pre-auricular points) and head shape, using a minimum of 60 face and scalp points. Three head position indicator coils were fixed to the participants’ head and referenced to the other digitized landmark, to localize the head’s position with the MEG system at the beginning of each block. Two EOG electrodes were placed above and below the left eye to record eye and blink movements. Two electrodes were placed across the plane of the chest to collect electrocardiographic (ECG) signals. Data were sampled at 2.4 kHz.

### Individual Retinotopic atlas from fMRI

MEG source analyses were constrained to each participant’s anatomy and retinotopically (functionally) defined regions of interest (ROIs). These ROIs were derived from fMRI results obtained in the same participants for other studies^16^. Volume segmentation of structural T1 MRI was performed with Freesurfer^17^, fMRI data were analyzed using mrVista^18^ to obtain population receptive field maps, using methods described in Dumoulin and Wandell^19^ and Clavagnier *et al*.^16^. The borders of the cortical area V1 were identified for every subject based on the location of visual meridians^20^. This region (V1) was imported into FreeSurfer as a custom atlas for subsequent MEG source analysis in Brainstorm^21^,^22^.

The high-resolution cortical surfaces (~160,000 vertices) were down-sampled to 15,000 vertices, to serve as image supports for cortically-constrained, distributed MEG source imaging^23^.

### Data preprocessing

Data preprocessing and data analysis were done using Brainstorm. The preprocessing steps consisted of detecting and attenuating artifactual contributions from heartbeats and eye blinks/movements to the MEG traces^24^. Occurrence of eye blinks and heartbeats were automatically detected from the EOG and ECG recordings. Signal-space projection vectors were then calculated for each type of artifact^25^, and in most cases the principal component with the highest eigenvalues was rejected for each artifact type. The data were then resampled to 1000 Hz; no band-pass filtering was performed.

### MEG source reconstruction

Noise covariance across MEG channels was estimated from the empty-room recording from each session. These noise statistics were used for the estimation of cortical currents with the depth-weighted L2-minimum norm estimator^23^. Source analysis yielded a linear kernel applied to MEG sensor data, to obtain MEG source time series at each of the vertices of the subjects’ cortical surface. Individually delineated primary visual cortex was used for analysis.

### Data analysis

Power spectral density (PSD) of MEG source time series was computed for all trials from 0.5 s to 4 s across all vertices (1000 ms window with 50% overlap) of primary visual cortex. Signals in the first 500 ms were removed to allow for establishment of the steady state. Responses in each block (n = 20) were standardized by dividing by the average responses to the mean luminance stimulus (response to 0% contrast stimulus) at the same frequency for each block, and subsequently averaged per block.

We combined the pre-1, pre-2 and pre-3 blocks together, resulting in only one power value for each of the four conditions (monocular 4 Hz, monocular 6 Hz, dichoptic and mean luminance responses) in the pre-patching session. In order to explore the effect of monocular deprivation on binocular interactions and to account for the non-stationarity of the response over the total experimental session, we defined a deprivation index as the ratio of the dichoptic responses divided by the monocular responses

### Phase analysis

The fast Fourier transform (FFT) was used to estimate the phase of SSVER signals at each vertex in V1 at 4 and 6 Hz over the 0.5 s–4 s time window, for each trial. The source location responding with maximum power at the stimulation frequency over all trials was identified and selected. The average of the phase angle at the tagged frequency across trials was then calculated. The average values of phase angle at 4 Hz (non-patched eye) and 6 Hz (patched eye) were compared between pre- and post- patching sessions. All phase data analysis was performed using the circular statistics toolbox in Matlab^26^.

## Results

**Figure 2:**
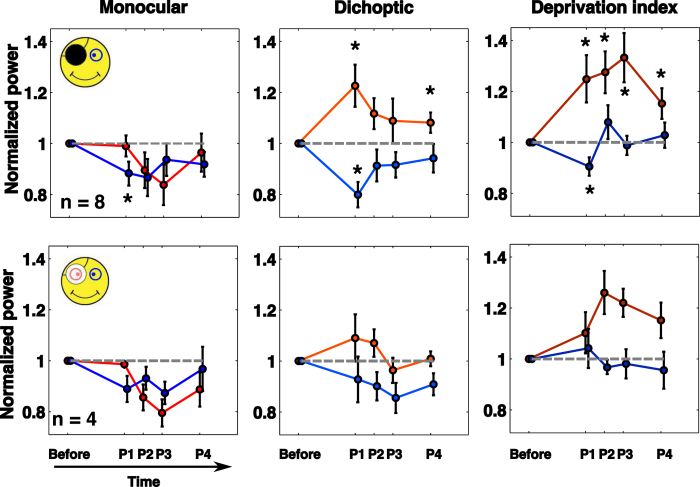
Effects of monocular deprivation. MEG power data for dominant (blue-patched eye) and non-dominant (red- unpatched eye) eyes before and after 150 min of monocular occlusion with either a black patch (top row) or a translucent patch (bottom row). Results are normalized to the pre-patching baseline (unity) and displayed for four time points after removal of the patch. Left column: monocular responses. Middle column: dichoptic responses. Right column: deprivation index.

Figure 2 displays the power data obtained for left (blue- patched eye) and right (red- unpatched eye) eyes using the frequency-tagging method normalized to the baseline power before monocular deprivation. The results after removal of the patch (top row black patch; bottom row translucent patch) are shown for 4 time points (P1 to P4). The first column represents the monocular signals (where only one eye was stimulated, the other eye seeing a mean luminance), the second column, the dichoptic interactions (where both eyes are stimulated simultaneously) and the third column, the deprivation index that corresponds to the dichoptic signal normalized by the monocular contributions. With a black patch (Fig. 2, top row), there is little monocular effect of patching, the signal for both eyes change in the same direction indicating that responsivity declined over time after patching. This was not unexpected because the deprivation itself lasted 2.5 hrs and MEG signals are known to vary spontaneously over time. The results of the dichoptic interaction signals are more noteworthy because the results after patching show a reciprocal change with an increase in sensitivity for the previously patched eye and a decrement in sensitivity for the unpatched eye (results for the P1 time point are statistically different from unity; one-tailed Wilcoxon signed-rank; 6 Hz: p = 0.0273; 4 Hz: p = 0.0078). There is a trend for this initial effect to decrease over time. The deprivation index in the third column has been normalized by the monocular responses to account for the time-dependent drift in sensitivity described above. Here we see the same trends, with the non-patched eye losing contribution (the first time point after patch removal is different from unity, 4 Hz in P1: p = 0.0391: corrected for multiple comparisons). The patched eye shows an improved contribution at all time points (6 Hz: P1: p = 0.0078; P2: p = 0.0078; P3: p = 0.0117; P4: p = 0.0391) which are significant and remain significant after passing the Benjamini Hochberg procedure for multiple comparisons.

The phase analysis of the data revealed no phase difference between pre and post patching recording sessions for either eye; for the patched eye (6 Hz), the difference between the pre- and post- patch phase was −0.26 + /− 3.83 ms (p = 0.9375 paired Wilcoxon signed-rank test); for the non-patched eye (4 Hz), the difference between the pre- and post- patch phase was 0.94 + /− 5.26 ms (p = 0.6875 paired Wilcoxon signed-rank test).

Previous psychophysical results comparing the effects of monocular deprivation with a black patch or with a translucent patch, have found no significant difference between these two forms of deprivation^3^. Hence we wanted to confirm these observations with MEG on a subset of participants. Indeed, the results using the translucent patch show the same trends as those for the black patch but did not reach significance.

## Discussion

Psychophysically, 2.5 hours of monocular occlusion produce a reciprocal change in visual threshold sensitivity measured psychophysically; the patched eye becoming more sensitive and the unpatched eye, less sensitive^3^. There is also a change in ocular dominance for suprathreshold stimuli that can be demonstrated either by the change in binocular rivalry^2^,^27^ or binocular combination^3^,^5^. A recent SSVER study showed that the signals from the previously patched eye increase in amplitude but no evidence was found to demonstrate any change in signals from the unpatched eye. The increase in amplitude in the patched eye was present for monocular as well as dichoptic stimulation^28^. Lunghi *et al*.^29^ were able to demonstrate a reciprocal effect for both eyes in their visually evoked potentials (VEP) EEG study. This group reported an increase of 66% in the early C1 component of the response for the deprived eye and a drop of 29% for the non-deprived eye. The source estimation placed the origin of the signal toV1 and concluded it was monocular in origin.

MEG provides a number of advantages over EEG, including a better source localization and minimal signal distortion. Our MEG results mirror the human psychophysics in that the patched eye increases in sensitivity and the unpatched eye reduces in sensitivity once the patch is removed. The response decrement in the unpatched eye is less than the response increment seen in the patched eye and this may be why it was not detected it in our previous SSVER EEG study^28^.

These results suggest that ocular dominance plasticity that occurs as a result of short-term monocular deprivation involves specifically contralateral interactions and can be understood in terms of our current model of binocular interaction in which contralateral inhibition regulates the contrast gain of each eye’s response prior to binocular combination^6^,^7^. This reciprocal change in monocular contrast gain has the consequence of unbalancing signals that are subsequently summed and hence altering ocular dominance in favor of the previously patched eye. These results can be directly related to the perceptual changes that are known to occur after short term monocular deprivation, for example, there is recent psychophysical support for interocular suppressive balance being altered by monocular patching^10^. It also provides an explanation for why the ocular dominance changes that result from short-term deprivation can be shown both for stimuli that can’t be fused, using binocular rivalry, and stimuli that can be fused using binocular combination paradigms^3^. The former is related to interocular inhibitory interactions, possibly related to dichoptic masking^8^,^9^ whereas the latter reflects the degree of excitatory combination that will be dependent on the strengths of the left and right eye inputs after their gain control by contralateral inhibition.

## Additional Information

**How to cite this article**: Chadnova, E. *et al*. Short-term monocular occlusion produces changes in ocular dominance by a reciprocal modulation of interocular inhibition. *Sci. Rep.* **7**, 41747; doi: 10.1038/srep41747 (2017).

**Publisher's note:** Springer Nature remains neutral with regard to jurisdictional claims in published maps and institutional affiliations.
