# Author Correction: Short-term monocular occlusion produces changes in ocular dominance by a reciprocal modulation of interocular inhibition

**DOI:** 10.1038/s41598-021-86898-5

**Published:** 2021-03-30

**Authors:** Eva Chadnova, Alexandre Reynaud, Simon Clavagnier, Robert F. Hess

**Affiliations:** Dept. Ophthalmology, McGill Vision Research, McGill University, Montreal, QC Canada

Correction to: *Scientific Reports* 10.1038/srep41747, published online 02 February 2017

In this Article, the legend of Figure 2 is incorrect:

“MEG power data for dominant (blue-patched eye) and non-dominant (red- unpatched eye) eyes before and after 150 min of monocular occlusion with either a black patch (top row) or a translucent patch (bottom row).”

should read:

“MEG power data for non-dominant (blue- unpatched eye) and dominant (red- patched eye) eyes before and after 150 min of monocular occlusion with either a black patch (top row) or a translucent patch (bottom row).”

In the Results section,

“Figure 2 displays the power data obtained for left (blue- patched eye) and right (red- unpatched eye) eyes using the frequency-tagging method normalized to the baseline power before monocular deprivation.”

should read:

“Figure 2 displays the power data obtained for non-dominant (blue- unpatched eye) and dominant (red- patched eye) eyes using the frequency-tagging method normalized to the baseline power before monocular deprivation.”

